# Concluding remarks: Atmospheric chemistry in cold environments†

**DOI:** 10.1039/d5fd00042d

**Published:** 2025-04-15

**Authors:** Markus Ammann

**Affiliations:** a PSI Center for Energy and Environmental Sciences, Paul Scherrer Institute 5232 Villigen Switzerland markus.ammann@psi.ch

## Abstract

Atmospheric chemistry in cold environments refers to key chemical processes occurring in Earth’s atmosphere in locations relevant for society including the polar areas, the free and upper troposphere, and the stratosphere. Atmospheric chemistry in these areas is relevant for local air quality, ecosystem health, regional and global climate. This *Faraday Discussion* comprised excellent coverage of these areas in terms of longitude and latitude, altitude and temperature. It also featured a broad coverage of disciplines between physical, analytical and theoretical chemistry and also the related fields covering aspects of biology, health, meteorology, social sciences and even including policy and economic aspects. A core aspect of the discussions was rooted in interfacing the related diverse competences. Because traditional atmospheric chemistry has evolved around knowledge of mechanisms and kinetics of chemical reactions first in the gas phase and later including condensed phases of aerosol particles and ground surfaces centering around room temperature, the speciality of relevance in this *Faraday Discussion* was the recent progress in better understanding the evolution of multiphase chemistry at low temperatures, where many relevant properties such as solubility and volatility change dramatically. This was embedded in discussions of the results and challenges of the most recent measurements from a range of campaigns and long-term observations at research stations. The discussion evolved around the chemical cycles of important trace constituents, the formation and evolution of particulate matter under cold conditions, the link between cloud glaciation and air-mass characteristics, air-quality in cold urban environments, biosphere–atmosphere interactions in a warming Arctic, but also the role of interfacial chemistry and reactivity as they are involved in multiphase chemistry processes. Future threats for the cold part of our atmosphere come from increasing human activities in both polar regions with their impacts on ecosystems, air quality and broader scale atmospheric composition as well as from discussions of geoengineering *via* solar radiation modification by stratospheric aerosol injection.

## Abstract 2

This paper summarizes the meeting on Atmospheric Chemistry in Cold Environments held in London. It puts the meeting into the context of this rapidly evolving scientific field and provides perspectives based on the discussions. Photo credit: K. Jacot.

## Graphical abstract

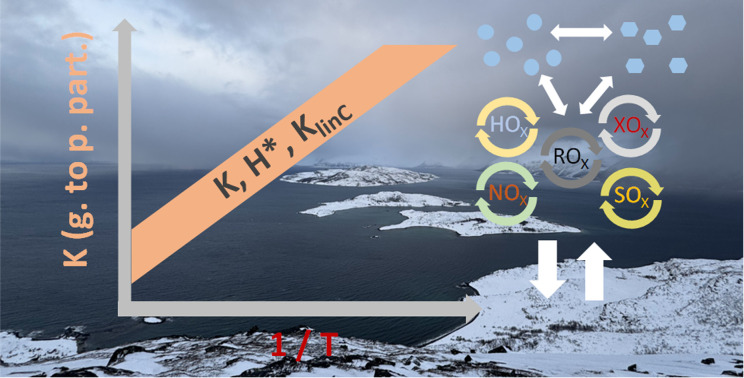

## Historic remarks

Interest in atmospheric chemistry in cold regions of the lower troposphere started with the discovery of ozone depletion events in the polar marine boundary layer and with measurements of elevated levels of nitrogen oxides and oxidised volatile organic compounds above sunlit snowpacks, for instance on the Greenland ice sheets.^1,2^ The International Polar Year 2007–2008 was a large, coordinated suite of polar observations, research, and analysis, expanding the knowledge base built on the legacies of previous polar studies.^3^ Three workshops were held specifically focusing on air–ice chemical interactions, 2006 in Grenoble (France), 2008 in Cambridge (UK) and 2011 in New York (USA) sponsored by the Air–Ice Chemical Interactions (AICI) and Halogens in the Troposphere (HitT) tasks of the International Global Atmospheric Chemistry (IGAC) project. These workshops attempted at gathering both the field measuring community active in polar environments but also colleagues from the laboratory, theory and atmospheric modelling community to obtain a more comprehensive understanding of polar (mostly boundary layer) atmospheric chemistry. The state of knowledge was outlined in a series of review articles initiated at these workshops.^4–15^ Joining forces between IGAC and the International Surface Ocean – Lower Atmosphere Study (SOLAS) projects and the International Arctic Science Committee (IASC), the Cryosphere and Atmospheric CHemistry (CATCH) initiative was launched. It aims at fostering multidisciplinary research on interactions between chemistry, biology, and physics within the coupled cryosphere–atmosphere system.^16^

Driven by the well-documented accelerated warming of the Arctic,^17^ interest in the not well-understood interplay between chemical and physical processes on the ground and in the air with climate forcers and oxidant budget led to a number of major field campaigns, which also had atmospheric chemistry topics in their portfolio, *e.g.*, ACE-SPACE (Antarctic Circumnavigation Expedition: Study of Preindustrial-like Aerosols and their Climate Effects),^18^ MOSAiC (Multidisciplinary drifting Observatory for the Study of Arctic Climate),^19^ ALPACA (Alaskan Layered Pollution and Chemical Analysis),^20^ ARTofMELT (Atmospheric rivers and the onset of Arctic melt),^21^ or CHACHA (CHemistry in the Arctic: Clouds, Halogens, and Aerosols).^22^

## Scope of this Discussion

Many aspects of atmospheric chemistry in polar regions are indeed linked to the low temperature on its own, although many peculiarities of boundary layer meteorology, cloud formation, the seasonal presence of sea-ice and snowpacks, the local marine and terrestrial biosphere or transport patterns of air masses from the lower latitudes create the particular environment for the chemical signatures, as summarized in the review series mentioned above. Along with the recognition of the vulnerability of Arctic and Antarctic regions came also the rise of interest in the so-called third pole on Earth, first coined for the Tibetan Plateau,^23^ but also more generally encompassing high alpine regions hosting the remaining mid-latitude glaciers and snow-cover. Measurements of atmospheric constituents in high-alpine research stations probing the free or upper troposphere have become an indispensable tool to document the state of the background atmosphere.^24,25^ Convective clouds are a major transport vehicle of air masses from the lower troposphere, and understanding those in terms of physical and chemical processes has become a major endeavour.^26^ The coldest lower atmospheric temperatures are found in the tropical tropopause layer, the entry point for transport from the troposphere to the stratosphere, and in the winter polar vortex over the poles. From a chemical point of view the stratospheric ozone layer is likely one of the most crucial features of the atmosphere. Its damaging through man-made chlorofluorocarbons (CFCs)^27^ has initiated major global coordinated and successful action for its protection, the results of which we are just about starting to witness in terms of its slow recovery.^28^ While the polar boundary layer, the midlatitude upper troposphere and the stratosphere share the temperature range, these vulnerable regions experience changes and threats driven by the warming climate, by the changing atmospheric composition, changing human activities and by the response of our society in terms of mitigation, adaptation and policy development. Overall, this widened the scope of atmospheric chemistry in cold environments and has set the scene for this *Faraday Discussion*.

**Fig. 1:**
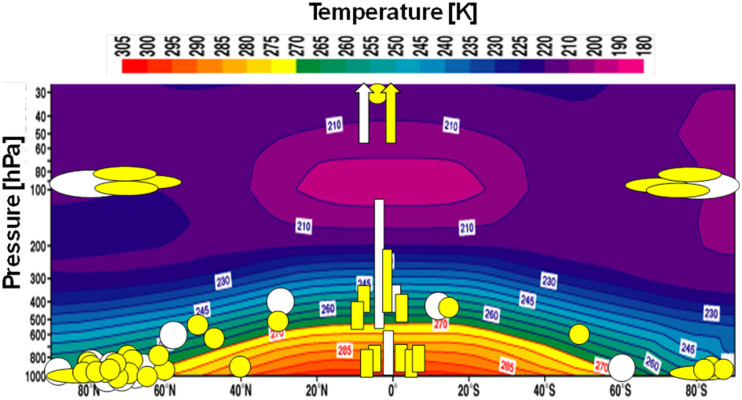
Studies presented at this Discussion mapped on the background of the zonal mean annual mean temperature (credit: ECMWF, downloaded from https://sites.ecmwf.int/era/40-atlas/docs/section_D25/charts/D30_XS_YEA.html) as a function of the altitude expressed as pressure. Round shapes represent studies addressing a specific location, while the rectangles gathered around 0° latitude refer to laboratory or theoretical work with the extent along the vertical axis reflecting their temperature range. White and yellow shapes refer to articles discussed and posters presented, respectively.

Fig. 1 shows the zonal mean temperature in Earth’s atmosphere, and the studies presented at this Discussion located at their respective latitude and altitude/temperature coordinates. Apart from covering the relevant temperature and altitude ranges, this Discussion was a perfect example of the interdisciplinary and integrative character of methods and approaches. A significant fraction of the articles circle around the major field campaigns ALPACA, CHACHA, ARTofMELT, MOSAiC and others, encompassing local measurements, ship cruises, aircraft campaigns and combinations therefrom. The value of the longterm efforts based on research infrastructure in the Arctic, Antarctica, in the Alps, in the Tibetan Plateau and in the Andes was clearly highlighted. Although not a strong focus at this Discussion, some authors mentioned the use of ice cores as archives for connecting records of past atmospheric chemistry signatures with historic and present day instrumental records. Instrumental analytical chemistry is a key aspect for both online and offline measurements, and several contributions dealt with innovation in this area. A broad range of techniques were employed including novel mass spectrometry tools, advanced optical spectroscopy and remote sensing methods, but also the use of low-cost sensors was discussed. Also in the laboratory work, the current challenges require novel surface sensitive tools such as photoelectron spectroscopy, infrared and non-linear optical spectroscopy, and neutron reflectivity and their development towards *in situ* measurement capabilities for low temperature systems. Especially lab work is ideally combined with theory support for electronic structure to support spectroscopy, reactivity of emerging species and molecular dynamics, where also new tools based on graph theory were presented. Dealing with such complex environments over so many scales requires appropriate modelling tools, and the work presented at this Discussion was accompanied by modelling at the local box model level, by regional and global chemistry transport modelling, and Earth system modelling, with focus in both the troposphere and the stratosphere.

## Topics in cold atmospheric chemistry

As mentioned above, the initial interest in chemical processes in polar regions circled about the episodic ozone depletion events (ODE)^8^ and the emission of nitrogen oxides and organics from sunlit snowpacks.^7^ Related to the first, understanding bromine chemistry has been a major focus from the beginning and remains an important aspect especially with respect to its link with nitrogen oxides from increasing local sources (https://doi.org/10.1039/D4FD00164H, https://doi.org/10.1039/D4FD00166D). Related, iodine chemistry has received increasing attention due its global importance including the stratosphere (https://doi.org/10.1039/D4FD00178H). Iodine is also potentially relevant to help Arctic cloud formation *via* nucleation of iodine oxides.^29^ Volatile organic compounds (VOC) were originally considered in the photochemistry in snowpack.^7^ Now they become more broadly addressed in terms of both primary, mostly biogenic (marine and terrestrial) emissions (https://doi.org/10.1039/D4FD00168K), and secondary sources also involved with secondary organic aerosol material. This relates to the partitioning of semivolatile secondary organics (https://doi.org/10.1039/D4FD00175C), but not only in the context of polar regions but also the upper troposphere in the outflow of convective clouds (https://doi.org/10.1039/D4FD00179F). Sulfur chemistry has been revisited to a significant degree due to sulfate formation *via* organosulfur compounds that enhances the contribution of sulfate to the particulate matter burden in polar regions (https://doi.org/10.1039/D4FD00170B). Ozone remains an important topic due to its role as a greenhouse gas. In the troposphere, the question arises to what degree the regional chemistry in the polar boundary layer affects ozone more broadly, *via* the export of ozone depleting halogens (https://doi.org/10.1039/D4FD00166D). In the stratosphere, the complex response of dynamics and chemistry to changing climate or for instance to changes in water vapor (https://doi.org/10.1039/D4FD00163J) are discussed at a time the first signs of recovery of the ozone layer, both mid-latitude and polar, from the reduction of CFCs is on the horizon.

Water vapor is certainly a key driver of atmospheric chemistry. It is a source of the hydroxyl radical (OH) that drives oxidation and ozone formation in the troposphere and depletion of ozone in the stratosphere. The water vapor partial pressure is tightly linked to the temperature in the atmosphere as it does not exceed saturation over larger spatial or temporal scales.^30^ The cold tropopause efficiently freezes out water before air-masses move into the stratosphere, keeping the latter dry, apart from chemical formation from the oxidation of methane. Apart from its contributions to the chemical cycles in the gas phase, water is the main actor in multiphase processes. It determines the structure and sea–air exchange properties of sea-ice (https://doi.org/10.1039/D4FD00172A). The water activity of aerosol particles controls their mixing state, phase behavior and viscosity (https://doi.org/10.1039/D4FD00175C). It is implemented in the glaciation of clouds (https://doi.org/10.1039/D5FD00005J). It is key in processes in convective cloud outflows (https://doi.org/10.1039/D4FD00179F). It is involved in new particle formation at high altitude (https://doi.org/10.1039/D4FD00171K) and in their activation to cloud droplets (https://doi.org/10.1039/D4FD00162A). Water is a driver of stratospheric temperature and dynamics (https://doi.org/10.1039/D4FD00163J) and of many more processes and phenomena. The peculiar properties of condensed water (liquid or ice) bring about many fundamental aspects of ion hydration,^31^ of structure at interfaces and its consequences.^32^ This relates to the interaction of organic monolayers at the liquid water surface (https://doi.org/10.1039/D4FD00167B) and to interfacial water at the onset of heterogeneous freezing (https://doi.org/10.1039/D4FD00165F). Last but not least, the elusive quasi-liquid layer on ice^11^ plays an important role for chemistry on ice particles or in the snow-pack (https://doi.org/10.1039/D4FD00161C, https://doi.org/10.1039/D4FD00169A, https://doi.org/10.1039/D4FD00157E, https://doi.org/10.1039/D4FD00176A).

At the larger scale the intimate coupling between the chemical cycles and the physical reality of phases raise topics such as biosphere–atmosphere interactions (*e.g.*, *via* ice nucleating biological particles (https://doi.org/10.1039/D4FD00160E) or VOC emissions (https://doi.org/10.1039/D4FD00168K)) or, when society meets the cold, air-quality in cold urban environments (https://doi.org/10.1039/D4FD00158C, https://doi.org/10.1039/D4FD00177J).

This overview over the topics covered at this *Faraday Discussion* makes it clear that only a system perspective going beyond the scientific disciplinary boundaries allows progress in understanding. Still, chemistry can remain an important and scientifically rewarding partial focus as clearly manifested in this Discussion.

## Aspects of cold atmospheric chemistry

As obvious from the brief mentioning of topics, issues and connections above, atmospheric chemistry in these environments is very complex. It is not only a simple set of gas phase reactions driving for instance much of ozone production from nitrogen oxides and volatile organic compounds in the warmer part of the lower troposphere. The atmosphere is a multiphase system involving condensed phases on the ground (ocean, sea-ice, snow, terrestrial surfaces, the urban technosphere) and in the air as aerosol particles, liquid and frozen cloud droplets or cirrus ice particles.^33^ This multiphase system is strongly interconnected chemically and physically.

**Fig. 2:**
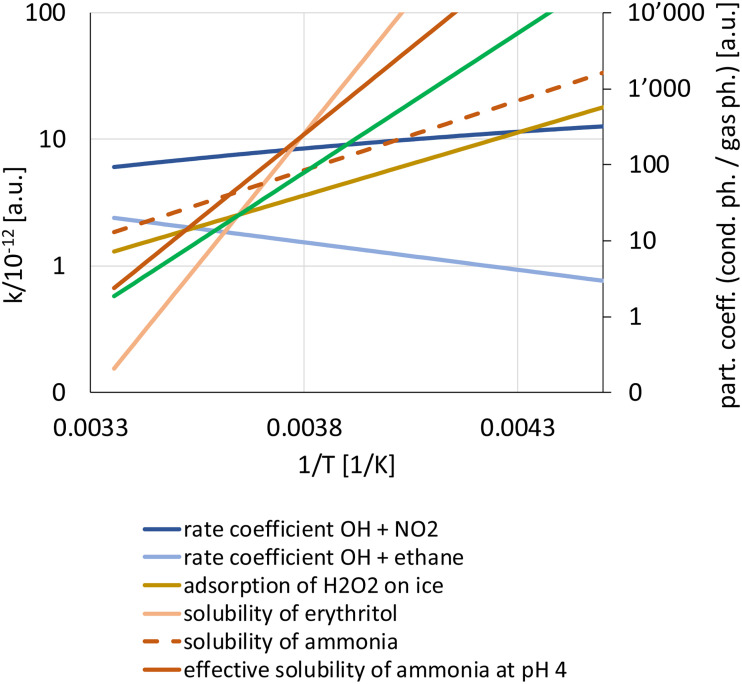
Temperature dependence of rate coefficients of gas phase reactions (blue, left *y*-axis) and partition coefficient for condensed phase–gas phase equilibria (right *y*-axis): solubility (red), adsorption on ice (dark yellow), volatility of an organic (green). All parameters scaled to emphasize differences in temperature dependence.

Emphasising the cold environments obviously alludes to atmospheric chemistry being different at low temperature than at around room temperature. In the gas phase, thermally activated reactions tend to slow down with lowering temperature and show positive temperature dependence, thus a decreasing slope of *k* as a function of 1/*T* in Fig. 2 for the example of the reaction of OH with an alkane.^34^ This classical Arrhenius behaviour indeed describes a range of relevant reactions in the troposphere and the stratosphere. However, for many complex forming reactions, this is counteracted by the equilibrium favouring the stability of the intermediate complexes (involving intermolecular interactions) towards lower *T*, which overrides the positive *T* dependence of some of the thermally activated elementary reactions involved as well.^35^ This may thus lead to overall negative temperature dependence, displaying an increasing slope in Fig. 2 for the example of the reaction of OH with NO_2_.^36^

The role of intermolecular interactions driving the free energy evolution is also the basis for the often very strong negative *T* dependence of solubility. As an example, the solubility of erythritol,^37^ as a proxy for tetrols formed in isoprene oxidation, is included in Fig. 2. Such strong temperature dependence leads to efficient partitioning of tetrols to the aqueous phase during updrafts above tropical forests (https://doi.org/10.1039/D4FD00179F). The partitioning of acidic or basic gases to aqueous phases is strongly affected by pH. This is also shown in Fig. 2 for the case of ammonia. At pH 4, due to the strong negative temperature dependence of the acid dissociation constant of ammonium,^38^ the slope of the effective solubility is nearly double that of ammonia alone (https://doi.org/10.1039/D4FD00173G).^39^ Ammonia is the major atmospheric base neutralizing the often acidic products of atmospheric oxidation, and this behavior is contributing a lot to particulate matter mass in cold urban environments.^39^

Even though the formation of highly oxidized organic species in the gas phase tends to be slower, the decreased volatility of the products at lower temperature more than compensates for that and allows sustaining efficient nanoparticle growth in cold environments.^40^ As another example, the evolving composition of biomass burning organic aerosol (BBOA) depends strongly on the combined effects of dilution and temperature drop,^41^ which is especially relevant when BBOA reaches high altitudes or even the stratosphere.^42^ Predicting volatility of organic aerosol components based on molecular formula (and usually not structure) is challenging, and volatility measurements demonstrate inconsistency with predictions at low temperature (https://doi.org/10.1039/D4FD00175C). The volatility plotted in Fig. 2 corresponds to a compound with intermediate volatility with an enthalpy of vaporization of 70 kJ mol^−1^.

Partitioning of gases to ice can be described by Langmuir type adsorption (https://doi.org/10.1039/D4FD00169A, https://doi.org/10.1039/D4FD00157E) with a partitioning constant giving the increase of surface coverage with increasing gas phase concentration in the linear part of the adsorption isotherm. The example shown in Fig. 2 is for H_2_O_2_,^43,44^ which is then the basis for its migration into and out of a snowpack under wind-pumping and temperature cycling, which connects back to the early snowpack studies.^7^ The ice surface itself, with its own complexity around the quasi-liquid layer, is thus a substrate for adsorbing species to interact with each other or to undergo acid–base chemistry or hydrolysis (https://doi.org/10.1039/D4FD00161C). However, snow is not just porous ice, but also contains a lot of trace constituents. For the soluble components, thermodynamics control the concentration of aqueous patches either coating the ice–air interface or embedded in grain boundaries. But whether bulk aqueous chemistry or rather more interfacial chemistry governs this system, remains an open question that can be tackled by linking a snow chemistry model with observations (https://doi.org/10.1039/D4FD00176A).

Another aspect that lower temperatures bring about is related to aerosol microphysical properties. Relative humidity in the atmosphere, outside clouds, is typically below saturation.^30^ Therefore, aqueous particles are high solute strength aqueous solutions. The viscosity of the solution increases towards lower water activity. This is especially relevant for aqueous aerosol dominated by secondary organics. At the lower temperatures of the mid-latitude upper troposphere, these solutions often pass the glass transition temperatures, making SOA particles more resemble marbles than liquid droplets.^45,46^ This affects their equilibration time scales with gas phase species, slows down reaction rates and affects also their ability to act as ice nuclei.^47^

As obvious from the discussion of temperature dependent kinetics, phase partitioning and solution properties, detailed understanding and modelling require access to reliable data. Since close to 50 years the Task Group of Atmospheric Kinetic Data Evaluation, presently under the umbrella of the International Union of Pure and Applied Chemistry (IUPAC),^48,49^ provides evaluated data for the kinetics of atmospheric chemical reactions. This was first focussing on gas phase reactions,^34,36,50–53^ but since a decade also of multiphase reactions,^54,55^ including partitioning coefficients for ice surfaces. The most recent development is to include aqueous phase reactions. Evaluated data are continuously updated and available at a database operated by AERIS (https://iupac.aeris-data.fr/). Similar data are provided by NASA JPL.^56^ Thermodynamic models as a basis for assessment of solution composition, viscosity, pH and surface tension, as provided by the AIOMFAC,^57,58^ E-AIM,^59,60^ ISORROPIA^61,62^ and more models with different functionalities are invaluable tools. Databases, data evaluation and data provision are important components in the links between basic laboratory work and modelling at all scales.^63^

As discussed above, water vapor is an important driving force to control the water activity in aerosol particles and through that a lot of the multiphase chemistry. The vapor pressure of liquid water is higher than that of ice, which opens a phase space window for instance relevant to how the water activity of the aqueous phase patches in equilibrium with ice is constrained in snow, but also to define the supersaturation of liquid clouds with respect to ice. Since glaciation of clouds is critically important for precipitation formation or also the persistence of clouds, ice nucleation has remained one of the most investigated and debated topics in atmospheric sciences.^64–66^ The high free-energy barrier to nucleation of ice can be lowered by ice nuclei (IN) that have the ability to induce ordering of water molecules in their vicinity through different interaction options and initiate heterogeneous freezing.^67^ Depending on the nature of the IN, amorphous, crystalline, molecule, macromolecule, complex bacteria or biological cells, these detailed interactions are very diverse. The ordering of the water molecules for instance in the presence of alcohol groups becomes apparent in the connectivity of the hydrogen bonding network as returned by graph theory (https://doi.org/10.1039/D4FD00165F), but can also be directly identified experimentally *via* X-ray absorption spectroscopy.^68^ Biological macromolecules are key to relevant ice nucleating particles that drive freezing processes especially in polar regions, where other IN are less abundant or their aging has made them less active (https://doi.org/10.1039/D5FD00005J).

## Challenges ahead

The chemical cycles discussed here offer still a lot of challenges, especially the multiphase chemistry part. For sulfate, significant progress has been achieved to better quantify the contribution of organosulfates (https://doi.org/10.1039/D4FD00170B). Also for bromine, a lot of progress has been made in understanding the chemical mechanisms, transport of the precursors and export into the wider troposphere (https://doi.org/10.1039/D4FD00166D). In turn, for iodine, substantial uncertainties remain with respect to the exact pathways for its release from snow (https://doi.org/10.1039/D4FD00178H). More generally, while established chemistry describes oxidation of iodide to iodate through both gas and aqueous phase pathways,^69^ the occurrence of IO, HOI and I_2_ in the upper troposphere and lower stratosphere^70,71^ indicates rapid recycling from iodate through not yet well-characterized pathways.^72^ Such recycling may also be relevant for iodine deposition into surface snow (https://doi.org/10.1039/D4FD00178H), but also for new particle formation in the Arctic through iodine oxides that could be recycled and thereby establish a catalytic role of iodine in Arctic haze formation.^29^ This would require more detailed iodine measurements in snow packs but also more generally in aerosol to better constrain iodine emission fluxes and impacts on tropospheric ozone (https://doi.org/10.1039/D4FD00178H).

Better understanding the role of secondary organics in cold urban haze or as CCNs in polar cloud formation has remained an important topic (https://doi.org/10.1039/D4FD00162A). Related, new particle formation in the outflow of convective clouds^26^ or in high altitude mountain ranges^73^ defines still poorly constrained ‘aerosol factories’ (https://doi.org/10.1039/D4FD00171K) determining the state of the cold, background upper troposphere. This topic is of course not limited to organics, as for instance efficient pathways have been suggested to bring ammonia to the upper troposphere following ice particle evaporation.^74^

A lot of progress has been achieved in our understanding of the nucleation of ice both at the fundamental process level (https://doi.org/10.1039/D4FD00165F), but also at the statistical level in the form of a correlation between particle properties and composition with observed ice nucleation behavior (https://doi.org/10.1039/D4FD00160E). However, at the bigger scale, linking cloud glaciation observations, *e.g.*, *via* emerging remote sensing approaches,^75^ with detailed chemical air mass characterisation would help obtaining predictive capabilities (https://doi.org/10.1039/D5FD00005J). At the process level, studies focused on the physical aspects of how freezing is induced with a link to the chemical composition of the substrate or how solutes in aqueous solutions interfere with that.^66^ Only recently, a study has established that chemical processes may be associated with freezing: it had been long well established that during the process of freezing of an aqueous solution, separation of anions and cations may occur due to contrasting ability to become embedded in a growing ice crystal, leading to the Workman–Reynolds freezing potential^76^ relevant for cloud electrification. Now Song *et al.* demonstrate that the local electrical field at the ice–liquid interface is strong enough that OH radicals are generated that lead to the formation of H_2_O_2_.^77^ The relevance of this phenomenon for the chemistry in clouds during glaciation both in mid-latitude or polar regions requires further exploration.

The latter case brings back the many fundamental science aspects related to processes at interfaces. The elusive quasi-liquid layer on pure ice, postulated by Faraday,^78^ has accompanied the science of phenomena under environmentally relevant cold conditions. This especially also applies to the history associated with the community gathered at this Discussion.^11^ The debate around its thickness has silenced somewhat due to the advent of surface selective methods (https://doi.org/10.1039/D4FD00169A, https://doi.org/10.1039/D4FD00157E) and better understanding of their probing depth and on the other hand substantial improvements on the theoretical side.^79^ The main challenge currently is to assess the role of the interfacial domain of ice surfaces in the presence of soluble and insoluble species, as well as the chemical reactivity therein.^11^ Progress on that side will be essential to improve our capabilities to reliably model chemistry in snow or in ice clouds (https://doi.org/10.1039/D4FD00176A).

The accelerated warming in the Arctic^17^ brings along a range of effects due to increased exploration, tourism, resource exploitation and transport, which lead to increased emissions, to extension of existing urban areas and the development of new ones. Thus, air quality aspects, already a significant issue now for some densely populated areas, will be of increasing concern. The special boundary layer meterological conditions are prone to high levels of air-pollutants representing a health issue for the local population (https://doi.org/10.1039/D4FD00158C, https://doi.org/10.1039/D4FD00177J). The associated gases and particles change also atmospheric chemistry at larger scales, such as by providing new CCNs, new INPs or affecting halogen chemistry (https://doi.org/10.1039/D4FD00164H). This means that the local effects indirectly translate into larger scale effects of relevance to the radiative properties and oxidation capacity of the atmosphere. Therefore, air quality aspects will become an increasingly important challenge.

Again, at a similar system-level, the warming climate changes biosphere–atmosphere interactions (https://doi.org/10.1039/D4FD00179F, https://doi.org/10.1039/D4FD00173G). Biogenic emissions (https://doi.org/10.1039/D4FD00168K) contribute to the composition, chemical properties, cloud occurrence and properties of the upper troposphere over low- and mid-latitudes, but also in polar regions, where thawing permafrost, changing sea-ice extent, changing snow-cover, along with the related ecosystems responses will require substantial efforts for assessing impacts on atmospheric composition and feedbacks between biosphere and climate.

The warming climate also impacts the stratosphere. Increasing water vapor (https://doi.org/10.1039/D4FD00163J) may change the dynamics and chemistry, for instance in terms of how fast the ozone layer is going to recover with the expected decrease in CFCs.^80^ The increased convective activity in the troposphere is contributing to this. The increased occurrence of wildfires leads to influx of organic particles through pyrocumulonimbus clouds,^42^ the consequences of which on stratospheric chemistry remain to be assessed. There are also other threads on the horizon for the stratosphere. The likely delayed path of society towards decarbonization may lead to the consideration of solar radiation modification technologies, among others stratospheric aerosol injection (SAI).^81^ The injection of sulfur dioxide as precursor for sulfuric acid particles mimicking volcanic eruptions has raised significant concerns as to induce warming in the stratosphere and additional ozone depletion.^82,83^ Alternatively, solid particles have been considered, which may have the potential to reduce such side effects.^84^ However, the uncertainties are huge^85,86^ and a sound scientific basis needs to be established under international governance frameworks^87^ before further considering such options.

This Discussion, on the basis of atmospheric chemistry, presents a manifestation of the ongoing community efforts to improve our understanding of chemical processes in cold atmospheric environments with a focus on polar regions and the other atmospheric compartments to which they are linked. The rapid changes in polar regions affect biodiversity, human society and global climate, as documented in the 6th assessment report of the IPCC.^88^ Building on that, presently consultations (https://indico.psi.ch/event/15591/) are underway for the 5th International Polar Year 2032–2033 (https://ipy5.info/resources/), and this Discussion is clearly a strong basis for the atmospheric chemistry pillar for further planning.

## Conflicts of interest

There are no conflicts to declare.

## Supplementary Material

FD-258-D5FD00042D-s001

## Data Availability

Data underlying Fig. 2 along with how they were obtained and secondary sources are provided as ESI.†
