# Supplementary material for: Concluding remarks: Atmospheric chemistry in cold environments
Source: Faraday Discuss. 2025 Apr 15;258:597–613. doi: 10.1039/d5fd00042d (PMC12056704; doi:10.1039/d5fd00042d)
Supplement: FD-258-D5FD00042D-s001 [file FD-258-D5FD00042D-s001.pdf]

Supplementary Information (SI) for Faraday Discussions.
This journal is © The Royal Society of Chemistry 2025

parameter	$1/T$	T	[M]	k_1	$K_{linC,H2O2}$	k_2	H_1	H_{NH3}	$K_{a,NH3}$	$K_{w,H2O}$	$K_{a,NH4+}$	H_{NH3}^*	p_{sat}
unit	1/K	K	cm^{-3}	$cm^3 s^{-1}$	cm	$cm^3 s^{-1}$	$M atm^{-1}$	$M atm^{-1}$	M	M^2	M^{-1}	$M atm^{-1}$	Pa
comment			1	2	3	4	5	6	7	8	9	10	11
	3.36E-03	298	8.95E+18	6.10E+00	7.25E+00	2.41E+00	2.08E-01	1.32E+01	1.81E-05	1.01E-14	1.79E+09	2.36E+00	1.86E+00
	3.42E-03	292	9.13E+18	6.45E+00	9.42E+00	2.25E+00	6.27E-01	1.76E+01	1.74E-05	6.32E-15	2.75E+09	4.86E+00	3.33E+00
	3.47E-03	288	9.26E+18	6.70E+00	1.13E+01	2.14E+00	1.34E+00	2.16E+01	1.69E-05	4.55E-15	3.71E+09	7.99E+00	4.97E+00
	3.52E-03	284	9.39E+18	6.96E+00	1.36E+01	2.04E+00	2.93E+00	2.65E+01	1.62E-05	3.22E-15	5.04E+09	1.33E+01	7.50E+00
	3.57E-03	280	9.52E+18	7.23E+00	1.65E+01	1.94E+00	6.56E+00	3.27E+01	1.55E-05	2.25E-15	6.90E+09	2.26E+01	1.15E+01
	3.62E-03	276	9.66E+18	7.51E+00	2.00E+01	1.84E+00	1.50E+01	4.06E+01	1.48E-05	1.55E-15	9.55E+09	3.88E+01	1.77E+01
	3.68E-03	272	9.80E+18	7.81E+00	2.45E+01	1.75E+00	3.52E+01	5.08E+01	1.39E-05	1.05E-15	1.33E+10	6.77E+01	2.77E+01
	3.73E-03	268	9.95E+18	8.12E+00	3.02E+01	1.65E+00	8.47E+01	6.40E+01	1.30E-05	6.94E-16	1.88E+10	1.20E+02	4.40E+01
	3.79E-03	264	1.01E+19	8.44E+00	3.74E+01	1.56E+00	2.09E+02	8.11E+01	1.21E-05	4.52E-16	2.68E+10	2.17E+02	7.09E+01
	3.85E-03	260	1.03E+19	8.78E+00	4.67E+01	1.47E+00	5.32E+02	1.04E+02	1.12E-05	2.89E-16	3.86E+10	4.00E+02	1.16E+02
	3.91E-03	256	1.04E+19	9.13E+00	5.87E+01	1.39E+00	1.39E+03	1.33E+02	1.02E-05	1.81E-16	5.62E+10	7.50E+02	1.92E+02
	3.97E-03	252	1.06E+19	9.50E+00	7.43E+01	1.30E+00	3.75E+03	1.73E+02	9.20E-06	1.11E-16	8.29E+10	1.43E+03	3.24E+02
	4.03E-03	248	1.08E+19	9.88E+00	9.48E+01	1.22E+00	1.04E+04	2.26E+02	8.23E-06	6.65E-17	1.24E+11	2.80E+03	5.55E+02
	4.10E-03	244	1.09E+19	1.03E+01	1.22E+02	1.15E+00	3.01E+04	2.99E+02	7.28E-06	3.89E-17	1.87E+11	5.60E+03	9.68E+02
	4.17E-03	240	1.11E+19	1.07E+01	1.58E+02	1.07E+00	8.97E+04	3.98E+02	6.37E-06	2.22E-17	2.87E+11	1.14E+04	1.72E+03
	4.24E-03	236	1.13E+19	1.11E+01	2.07E+02	9.97E-01	2.78E+05	5.36E+02	5.51E-06	1.23E-17	4.47E+11	2.40E+04	3.12E+03
	4.31E-03	232	1.15E+19	1.15E+01	2.73E+02	9.27E-01	8.94E+05	7.28E+02	4.70E-06	6.65E-18	7.07E+11	5.15E+04	5.77E+03
	4.39E-03	228	1.17E+19	1.20E+01	3.63E+02	8.59E-01	3.00E+06	1.00E+03	3.96E-06	3.49E-18	1.13E+12	1.14E+05	1.09E+04
	4.46E-03	224	1.19E+19	1.24E+01	4.89E+02	7.94E-01	1.05E+07	1.39E+03	3.28E-06	1.77E-18	1.85E+12	2.58E+05	2.11E+04

Comments:

1: calculated by 101325/(3.8E-23*T)/1000000

2: OH + NO_2 + M; calculated by ((3.2E-30*(T/300)^-4.5)*[M]*(0.00000000003))/((3.2E-30*(T/300)^-4.5)*[M]+(0.00000000003))*10^(LOG10(0.41)/(1+(LOG10((3.2E-30*(T/300)^-4.5)* [M]/(0.00000000003))/(0.75-1.27*LOG10(0.41)))^2)); see https://iupac-aeris.ipsl.fr/datasheets/pdf/NOx13.pdf for details; scaled to fit plot

3: K_{linC} for H_2O_2 on ice; calculated by 0.000021*EXP(3800/T); see https://iupac-aeris.ipsl.fr/datasheets/pdf/HI5.pdf for details; scaled to fit plot

4: OH + C_2H_6; calculated by 0.0000000000069*EXP(-1000/T); see https://iupac-aeris.ipsl.fr/datasheets/pdf/HOx_VOC4.pdf for details; scaled to fit plot

5: solubility of erythritol, see https://henrys-law.org/henry/casrn/149-32-6, scaled to fit plot

6: solubility of ammonia, see https://henrys-law.org/henry/casrn/7664-41-7, scaled to fit plot

7: acid dissociation constant of ammonia, calculated by 0.00001805*EXP(-1.5*(298/T-1)+26.92*(1+LN(298/T)-298/T)), taken from Kim et al., https://doi.org/10.1080/02786829308959628, Table 3.

8: dissociation constant of water, calculated by 0.0000000000000101*EXP(-22.52*(298/T-1)+26.92*(1+LN(298/T)-298/T)), taken from Kim et al., https://doi.org/10.1080/02786829308959628, Table 3, scaled to fit plot.

9: $K_{a,NH4+} = K_{a,NH3} / K_{w,H2O}$

10: $H_{NH3}^{*} = H_{NH3} \times (1 + K_{a,NH4+} * (0.0001\ M))$

11: calculated by =0.000000000001*EXP((70000/8.314)/T), scaled to fit plot.
